# Luminescent and UV-Shielding ZnO Quantum Dots/Carboxymethylcellulose Sodium Nanocomposite Polymer Films

**DOI:** 10.3390/polym10101112

**Published:** 2018-10-08

**Authors:** Tianyi Li, Bin Li, Yali Ji, Lili Wang

**Affiliations:** College of Science, Northeast Forestry University, Harbin 150040, China; 18845562646@163.com (T.L.); libinrainkiss1014@163.com (B.L.); jyl1513062809@163.com (Y.J.)

**Keywords:** UV-shielding quantum dots, biomaterials, luminescent, polymer, nanocomposite

## Abstract

Luminescent and UV-shielding bio-polymers have seldom been reported. Zinc oxide quantum dots (ZnOQD) (~3.2 nm) was synthesized by a short-term sol-gel method. ZnOQD possessed a crystal lattice spacing of 0.28 nm and a hexagonal wurtzite structure. Luminescent and UV-shielding ZnOQD/carboxymethylcellulose sodium (CMC) nanocomposite polymer films were successfully fabricated by incorporating ZnOQD into a CMC matrix through a solution casting method. Thermal analysis demonstrated that the ZnOQD reduce the thermal decomposition rate of CMC, and a large number of ZnOQD can promote the catalytic degradation of ZnOQD/CMC nanocomposites. Furthermore, ZnOQD/CMC hybrid polymer films exhibited photoluminescence with maximum emission wavelength at 525 nm. More significantly, ZnOQD/CMC showed prominent UV-absorbing capability. Such ZnOQD/CMC nanocomposite polymer films are promising in UV-shielding and optical applications.

## 1. Introduction

Recently, many composites have been reported and have aroused current interest [1,2]. In particular, many natural bio-polymers have been widely used in packaging [3,4] and within the medical industry [5,6] due to their biocompatibility [7], biodegradability [8], low toxicity and edibility [9,10]. Carboxymethylcellulose sodium (CMC) as a biopolymer has high optical transparency, good chemical resistance and film forming properties. More importantly, CMC is rich sources and sustainable. However, luminescent and UV-shielding CMC-matrix nanocomposites have seldom been reported.

ZnO quantum dots (ZnOQD) with a wide band gap of 3.37 eV and a rather large exciton binding energy of 60 meV exhibit unique optical and electrical properties [11] due to the quantum confinement effect [12,13]. ZnOQD has the merits of being non-toxic [14], water dispersibility [15] and biocompatibility [16]. Therefore, in the present work, ZnOQD was synthesized using a short-term sol-gel method. The morphology, particle size, crystalline structure, and thermal and optical properties were characterized using an enlarged high-resolution transmission electron microscope (HRTEM), selected area electron diffraction (SAED), powder X-ray diffraction (XRD) pattern, thermogravimetric analysis, ultraviolet-visible (UV-vis) and photoluminescence spectra, respectively. Furthermore, to obtain CMC-based luminescent polymer hybrid films, ZnOQD/CMC nanocomposites were successfully prepared via a green simple procedure. The optical and UV-shielding properties of ZnOQD/CMC composites were investigated. This work broadened the applications of biopolymers in UV-shielding [17] and within the optical field.

## 2. Materials and Methods

### 2.1. Materials

Zinc acetate (99%), lithium hydroxide (99%) and carboxymethylcellulose sodium (CMC) (99%) were purchased from Tianjin Kermiou Chemical Reagent Co. Ltd. (Tianjin, China). Ethanol (99%) was purchased from Tianjin Fuyu Fine Chemical Co. Ltd. (Tianjin, China). All the reagents and chemicals were of analytical grade without further purification. Deionized water (Milli-Q Academic, Beijing, China) was used throughout the whole experiment.

### 2.2. Preparation of ZnOQD and ZnOQD/CMC Nanocomposites

Ethanol was chosen as solvent. Firstly, 0.878 g (4 mmol) of zinc acetate and 0.2672 g (6 mmol) of lithium hydroxide were dissolved in 100 mL of ethanol, respectively. Secondly, the above two solutions were ultrasonicated at 70 °C for 20 min. Thirdly, prior to the addition of lithium hydroxide solution, zinc acetate solution was refluxed at 78 °C for 15 min under continuous stirring. Then the mixed solution reacted by further refluxing with stirring under 78 °C for 25 min. The as-prepared zinc oxide quantum dots were dried in the oven at 70 °C for 2 h and the resulting sample was labeled as ZnOQD.

### 2.3. Fabrication of ZnOQD/CMC Composites

Three grams of CMC were dissolved in 100 mL of distilled water under stirring for 4 h at 60 °C. ZnOQD was dispersed in 20 mL of distilled water treated by ultrasonic for 1 h. Then ZnOQD and CMC aqueous solution were mixed. The mixtures were casted onto glass substrates with no post treatment. Furthermore, the glass substrates were transferred to the oven and dried for 10 h at 60 °C to obtain ZnOQD/CMC films. The ZnOQD/CMC composites with 5, 10 and 15 wt % loading of ZnOQD were fabricated, respectively. Thus, the resulting ZnOQD/CMC films were labeled as 5 wt % QD-CMC, 10 wt % QD-CMC and 15 wt % QD-CMC, respectively.

### 2.4. Characterization Techniques

The specimens were examined in a TECNAI 10 PHILIPS transmission electron microscope (TEM) (Amsterdam, Holland) operated at the accelerating voltage of 100 kV. The powder X-ray diffraction (XRD) pattern of the samples was recorded by a D/MAX 2200 diffractometer (Rigaku, Tokyo, Japan) using Cu Kα radiation in a scan step of 0.02° and a scan range between 5° and 70°. All thermogravimetric analysis (TGA) tests were carried out by a thermal analyzer (TGA pyris 1, Perkin Elmer Co., Waltham, MA, USA) at a linear heating rate of 10 °C/min under pure nitrogen within the temperature range of 50 to 800 °C. The mass of the samples was kept within 3–4 mg. Ultraviolet-visible (UV-vis) and photoluminescence spectra of ZnOQDs and ZnOQD/CMC nanocomposites were recorded on a GENESYS 10 UV-vis spectrometer (Beijing, China) and fluorescence F-4600 fluorescence spectro-photometer (Hitachi, Japan), respectively.

## 3. Results and Discussion

### 3.1. Morphology and Diffraction Analysis of ZnOQD 

**Figure 1:**
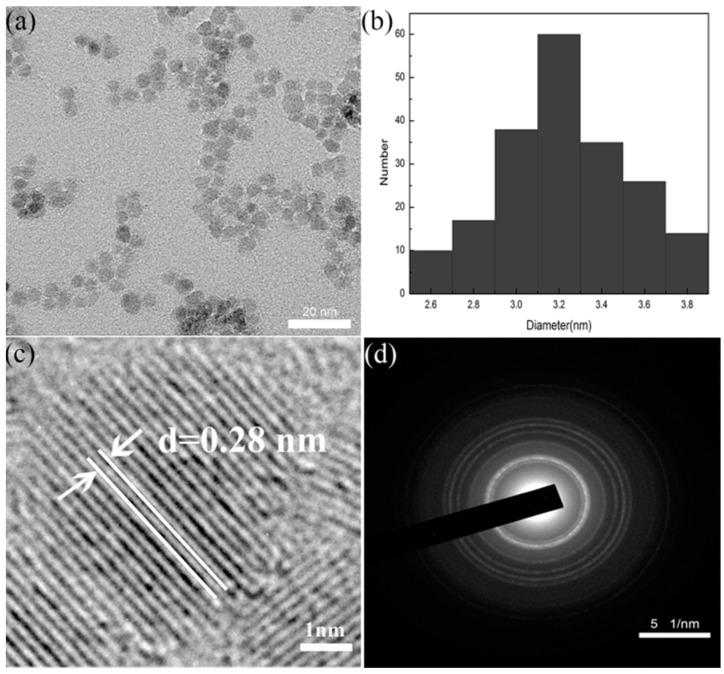
(**a**) TEM image of ZnOQD, (**b**) diameter distribution histogram calculated by TEM, (**c**) enlarged HRTEM (high-resolution transmission electron microscope) image of ZnOQD, (**d**) SAED (selected area electron diffraction) pattern of ZnOQD.

Figure 1a shows TEM images of ZnOQD featured spherical particles along with good monodispersity. Figure 1b displays the size distribution histogram measured from 100 particles. As presented, the average size of ZnOQD is (3.2 ± 0.2) nm. Figure 1c gives the enlarged high-resolution transmission electron microscope (HRTEM) of ZnOQD. The ZnOQD has excellent crystallinity due to the existence of a crystal lattice spacing of 0.28 nm [18]. Figure 1d shows the selected area electron diffraction (SAED) pattern of ZnOQD. The diffraction rings were referred to as (100), (002), (101), (102), (110), (103) and (112) planes, respectively, indicating that the ZnOQD featured a hexagonal wurtzite structure with high crystallinity [11].

### 3.2. XRD and Photoluminescence Analysis of ZnOQD 

**Figure 2:**
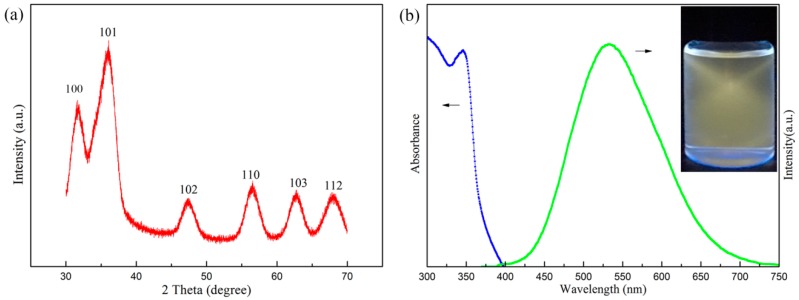
(**a**) XRD pattern of ZnOQD, (**b**) UV-vis and photoluminescence spectra of ZnOQD.

Figure 2a shows the XRD pattern of ZnOQD. From Figure 2a, it can be observed that the dominant peaks of ZnOQD appear at 2 *θ* values of 31.9°, 36.1°, 47.4° and 56.6°, corresponding to (100), (101), (102), and (110) planes, respectively. Figure 2b displays the UV-vis and photoluminescence spectra of ZnOQD, indicating that the ZnOQD featured good optical properties with a clearly resolved absorption peak at 350 nm and a symmetrical emission peak at 525 nm. The inset in Figure 2b is the digital photo of ZnOQD aqueous solution, which is excited under 365 nm UV lamp radiation. Correspondingly, the ZnOQD aqueous solution exhibits yellow fluorescence due to the quantum-size and surface defects [15].

### 3.3. Morphology Analysis of ZnOQD/CMC Composites

**Figure 3:**
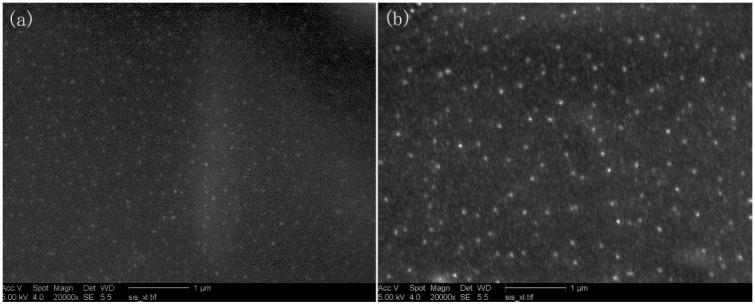
SEM images of (**a**): 5 wt % QD-CMC and (**b**): 15 wt % QD-CMC composites.

Figure 3a,b give the SEM images of ZnOQD/CMC composites with 5 and 15 wt % loading of ZnOQD, respectively. From Figure 3a, the CMC-based nanocomposite showed the uniform distribution of ZnOQD. As the ZnOQD content increased from 5 to 15 wt %, a large number of ZnOQD were observed in a 15 wt % QD-CMC composite, as shown in Figure 3b. Both 5 wt % QD-CMC and 15 wt % QD-CMC composites show homogeneous distribution of ZnOQD within the film matrix.

### 3.4. XRD Analysis of ZnOQD/CMC Composites

**Figure 4:**
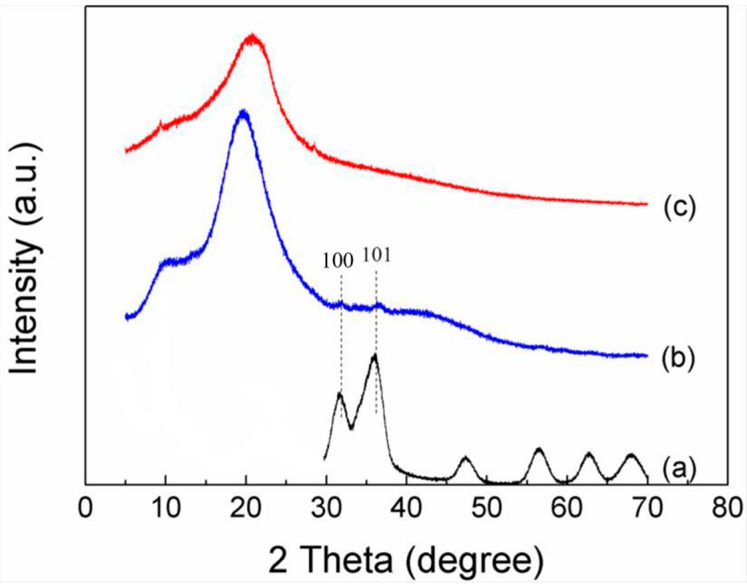
XRD patterns of (a): ZnOQD (b): 10 wt % QD-CMC composites and (c): pure CMC.

Figure 4 shows the XRD pattern of pure CMC and 10 wt % QD-CMC composites. Both CMC and 10 wt % QD-CMC composites exhibited the characteristic diffraction peaks of CMC at 2 *θ* values of 22.2°. Furthermore, a 10 wt % QD-CMC composite displays (100) and (101) diffraction peaks of ZnOQD at 2 *θ* values of 31.9° and 36.1°, respectively. XRD analysis of composites also confirmed that ZnOQD dispersed in the matrix of CMC, which is in accordance with SEM analysis.

### 3.5. Thermal Analysis of CMC and Composites

**Figure 5:**
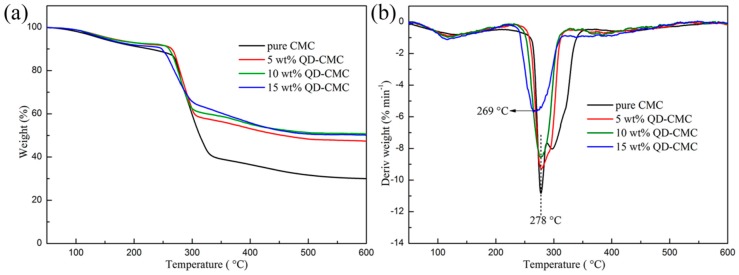
Thermogravimetric analysis (TGA) and differential thermogravimetry (DTG) curves of CMC and composites, (**a**) TGA curves; and (**b**) DTG curves.

Figure 5a,b illustrate the TGA and differential thermogravimetry (DTG) curves of CMC and its composites with 5, 10 and 15 wt % loading of ZnOQD, respectively. As shown in Figure 5a, the first stage with a small weight loss of around 100 °C corresponds to the evaporation of adsorbed water. The second stage with a very fast and significant weight loss, occurring at around 250–340 °C, is owing to the degradation of CMC. The third thermal degradation which started at around 300–380 °C is due to the pyrolysis of carbonaceous residues at high temperatures. Moreover, the char yield of 5–15 wt % QD-CMC composites is 42%, 40% and 36% at 600 °C, respectively. From Figure 5b, it can be seen that the maximum thermal degradation temperature of 5 wt % QD-CMC and 10 wt % QD-CMC composites were both at 278 °C, which was the same as that of pure CMC. The maximum thermal degradation temperature of a 15 wt % QD-CMC composite was at 269 °C, which was lower than pure CMC film. The reason is that when the ZnOQD loading increases to 15 wt %, a lot of metal and metal oxides promote the catalytic degradation of CMC on heating [19,20]. Furthermore, it is shown that the maximum thermal degradation rate gradually decreases with the increase of ZnOQD loading.

### 3.6. Photoluminescence Analysis of ZnOQD/CMC Composites 

**Figure 6:**
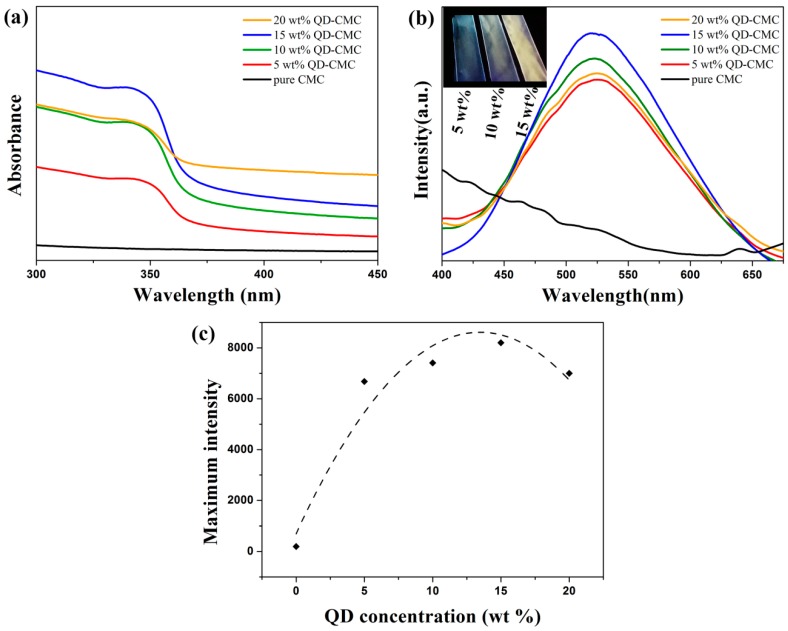
(**a**) UV-vis spectra and (**b**) photoluminescence spectra (ex = 350 nm) of pure CMC and composites. Inset in (**b**) is digital photographs of ZnOQD/CMC nanocomposite films under 365 nm UV lamp. (**c**) Maximum intensity of the photoluminescence spectra of ZnOQD/CMC nanocomposite films with different ZnOQD concentrations.

Figure 6a gives the UV-vis spectra of pure CMC and ZnOQD/CMC composites, respectively. Pure CMC cannot absorb UV radiation. However, the UV absorption band of ZnOQD/CMC almost covers the whole UV range of up to about 375 nm [21]. Figure 6b shows photoluminescence spectra of pure CMC and ZnOQD/CMC composites containing 5, 10, 15 and 20 wt % ZnOQD excited by 350 nm wavelength. It clearly exhibits that the pure CMC film features no optical properties. Comparatively, the ZnOQD/CMC composites present significant emission peaks. The maximum emission wavelength appears at 525 nm, which is in accordance with the previously reported ZnOQD [22]. Meanwhile, the results indicate that PL emission intensity is significantly enhanced by adding ZnOQD nanoparticles into a CMC polymer matrix, and a maximum PL emission intensity is reached by adding 15 wt % ZnOQD. The inset in Figure 6b is the digital group of photographs of 5 wt % QD-CMC, 10 wt % QD-CMC and 15 wt % QD-CMC composites in a dark box under 365 nm UV lamp irradiation. It is obvious that the fluorescent color of composites gradually matches ZnOQD solution with the ZnOQD concentrations increasing to 15 wt %. 

Figure 6c shows the maximum intensity of the photoluminescence spectra of ZnOQD/CMC nanocomposite films with different ZnOQD concentrations, indicating that the emission intensity does not increase linearly with the increasing loading amount of ZnOQD. It can be found that the emission intensity has an increasing tendency, with the concentration of ZnOQD ranging from 5 to 15 wt %. The obvious increase of emission intensity for a 15 wt % QD-CMC composite can be interpreted in two ways: Charge trapping effect and chain separation. The ZnOQD can trap electrons and allow more holes to recombine through the interface of CMC and ZnOQD. The exciton formation inside 15 wt % QD-CMC composite layers is increased in comparison with 5 wt % QD-CMC and 10 wt % QD-CMC composites, and thus enhances luminescent properties. However, the emission intensity of the 20 wt % QD-CMC composite is decreased significantly. A reason for this is that by adding too much ZnOQD, most electrons are mainly blocked around the surface of ZnOQD and restrict the recombination population for the ZnOQD/CMC composites [23]. On the other hand, after 15 wt %, PL emission is partially quenched due to the agglomeration of ZnOQD particles [1,23,24]. Therefore, in order to achieve the maximum output efficiency of the ZnOQD/CMC composites in terms of their luminescence levels, the recommended concentration range of ZnOQD is ~15 wt %.

## 4. Conclusions

In summary, ZnOQD was synthesized through a facile ultrasound assisted sol-gel method in a short time. The ZnOQD/CMC composite films were successfully fabricated via an environmentally friendly route. We demonstrated the good optical properties of ZnOQD/CMC composites containing 15 wt % ZnOQD. In particular, the ZnOQD/CMC composites can absorb the UV radiation of wavelengths less than 375 nm. Therefore, this work raised exciting opportunities of ZnOQD/CMC composites for UV-shielding and optical applications.
